# Cuticular microstructure of the locust femur–tibia joint

**DOI:** 10.1242/bio.061934

**Published:** 2025-07-31

**Authors:** Christoph Bruns, Vassileios Bekas, Susanna Labisch, Jan-Henning Dirks

**Affiliations:** ^1^ Biomimetics-Innovation-Centre, Hochschule Bremen - City University of Applied Sciences, 28199 Bremen, Germany; ^2^ Department of Biomimetics, Hochschule Bremen - City University of Applied Sciences, 28199 Bremen, Germany

**Keywords:** Exoskeleton, Articular membrane, Cuticular folding, Locust, Leg joint

## Abstract

In insect exoskeletons, articular membranes connect the sclerotized hard segments within joints, ensuring protection, mobility, and resilience to mechanical stresses. During exoskeletal movement, these membranes experience tensile and compressive forces, leading to either stretching or the formation of cuticular folds. The mechanisms underlying cuticular folding remain unclear, particularly whether folds are regular (specific) or irregular (non-specific) and how cuticle ultrastructure influences folding patterns. To address these questions, we examined the femur–tibia joints in the mesothoracic legs of locusts (*Locusta migratoria*) using non-destructive micro-CT, histological methods, and scanning electron microscopy. The joints were analyzed at different flexion angles and maturity stages to characterize membrane folding. Our findings reveal distinct scales of cuticular folds in the femur–tibia joint: macrofolds associated with internal structures such as muscle attachment sites and microfolds potentially linked to cuticle ultrastructure, surface properties, or membrane thickness differences.

## Teaser

**Summary:** Insect joint membranes form distinct macro- and microfolds during movement, shaped by internal structures and cuticle ultrastructure.

## INTRODUCTION

Insects have a remarkable exoskeleton composed of rigid sclerotized segments interconnected by flexible, soft membranes. The primary component of this structure, the cuticle, is the second most common biological composite material on Earth (Vincent, 2002). Insect cuticle is a highly complex composite material comprising chitin fibres embedded in a protein matrix. Its mechanical properties are influenced by a multitude of factors, including the ultrastructure and the orientation of the chitin fibres, water content, sclerotization and adaptations in micro- and nanostructure (Müller et al., 2008; Vincent, 1981; Vincent and Wegst, 2004; Taylor and Dirks, 2012; Chandran et al., 2016). Among the various regions of the exoskeleton, intersegmental and articular membranes play particularly significant roles due to their flexibility and distinct biomechanical properties (Hillerton, 1984).

Intersegmental membranes, such as those in the abdomen, and articular membranes, like those found in joints, differ substantially from sclerotized segments in their structure and function. Unlike the rigid cuticle of sclerotized segments, membranes are less sclerotized, allowing for greater flexibility and expansion (Vincent, 1981; Müller, 2009; Sun et al., 2021; Göttler et al., 2021; Silva et al., 2023). In the abdomen of female locusts, for instance, Vincent (1981) documented a combination of membrane stretching and folding during expansion of the abdomen for oviposition. Similarly, Zhao et al. (2016) described the folding morphology of intersegmental membranes in the telescoping abdomen of honeybees. However, despite these studies, the ultrastructure and functional adaptations of these membranes remain poorly understood. Folding mechanisms are also documented in other insect structures, such as wings, where folding contributes to compact storage and deployment (Faber et al., 2018; He et al., 2020).

In arthropods, articular membranes in the joints facilitate dynamic movements, as observed in jumping spiders (Blickhan and Barth, 1985; Göttler et al., 2021). However, key differences exist between spiders and insects in terms of joint mechanics. In spiders, leg extension is driven by internal haemolymph pressure, whereas in insects, muscle power predominates. Despite these distinctions, little is known about the behaviour of articular membranes in insect leg joints, particularly whether during movement the cuticle of these membranes undergoes stretching or folding. Previous studies have focused on abdominal membranes (e.g. Vincent, 1981; Hackman and Goldberg, 1987), which perform distinct functions such as expansion for feeding or reproduction. The limited attention given to articular membranes in insect joints highlights the need for a focused investigation of their morphology and movement.

This study aims to address these knowledge gaps by examining the articular membrane in the femorotibial joint of the mesothoracic leg in *Locusta migratoria*, a commonly used model organism in insect and cuticle biomechanics. We investigate whether the primary mechanism during joint movement is membrane stretching or folding, with initial findings suggesting that folding predominates. Furthermore, we explore the factors influencing fold formation, including potential local sclerotization, membrane ultrastructure, and surface properties. Specifically, we address the following questions: does the cuticle in the membrane stretch or fold? If present, are folds specific or non-specific? Are these folds affected by aging of the insect or usage of the legs? How does the histological composition of the articular membrane correlate with folding, and how does it differ from intersegmental membranes? Finally, we investigate the potential role of surface structures in fold formation.

To answer these questions, we employed a multidisciplinary approach. Non-destructive X-ray imaging was used to study membrane behaviour at different joint positions across developmental stages. Histological analyses, including staining techniques and light microscopy, were performed to examine membrane ultrastructure. Additionally, scanning electron microscopy was used to investigate surface properties. These methods provide a comprehensive understanding of the structure-function relationship in the articular membrane, offering new insights into the biomechanics of insect joints.

## RESULTS

### Cuticle folding in the articular membrane

The microCT results show the presence of a characteristic folding pattern in the articular membrane of the mesothoracic legs. Two types of folds with different length scales were identified: (1) one to two larger macrofolds in the distal area of the membrane, which slightly change depending on the joint angle; (2) numerous smaller fingerprint-like “microfolds” distributed across the entire articular membrane.

### Shape and kinematic of the macro folds

**Fig. 1.**
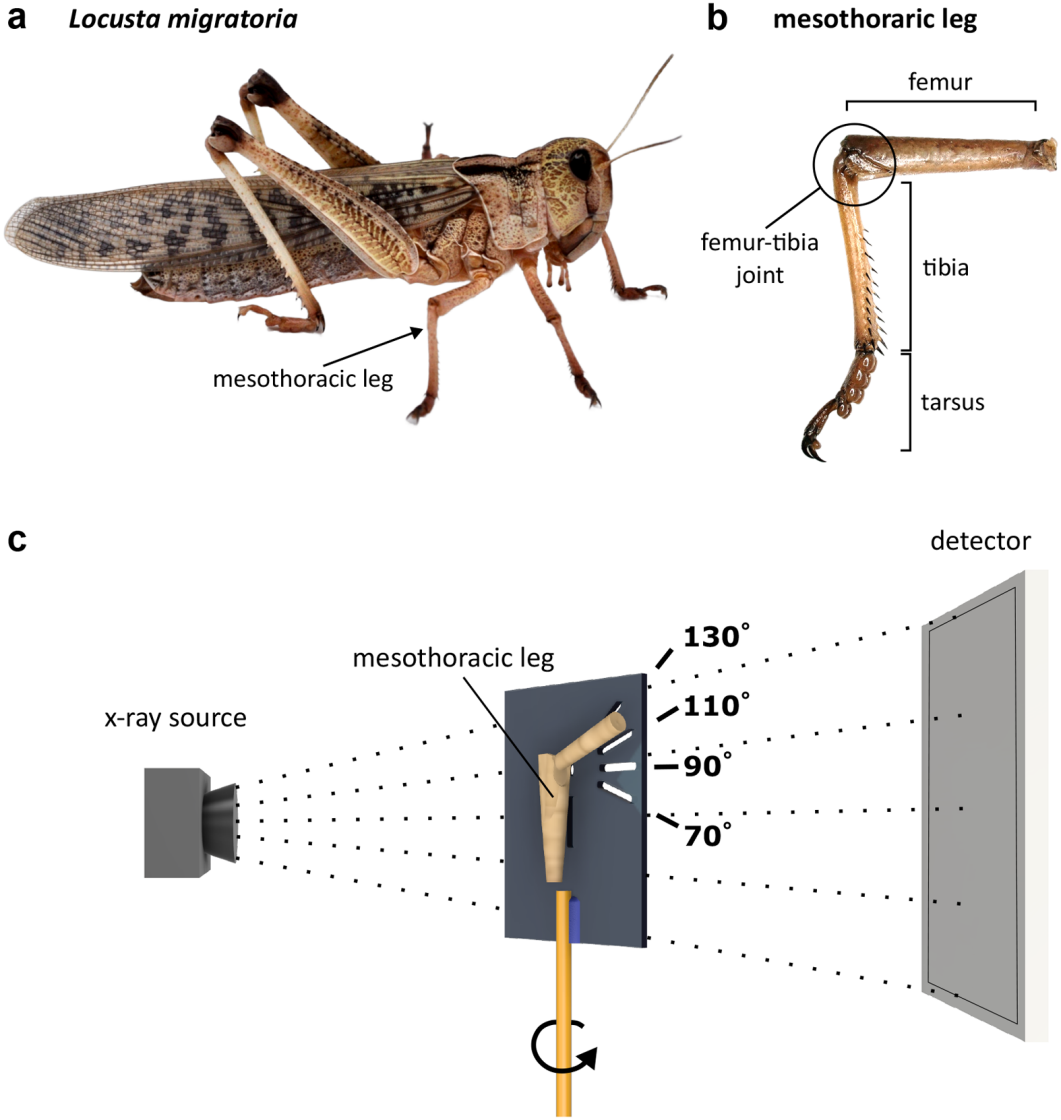
**Principle used for X-ray imaging of insect leg joints at predefined deflection angles.** (A) *Locusta migratoria* was used as a typical model organism. (B) The femur–tibia joints of the mesothoracic leg were used as representative samples for walking legs in insects. (C) The sample holder is made of acrylic glass (width: 10 mm, height: 20 mm, depth: 2 mm), with engraved markings at the angles of 70°, 90°, 110° and 130°. The acrylic glass is glued to a 3 mm brass rod, which can be clamped in the CT machine. The insect joint is attached to the acrylic glass with dental wax using the markings with the preferred joint position.

**Fig. 2.**
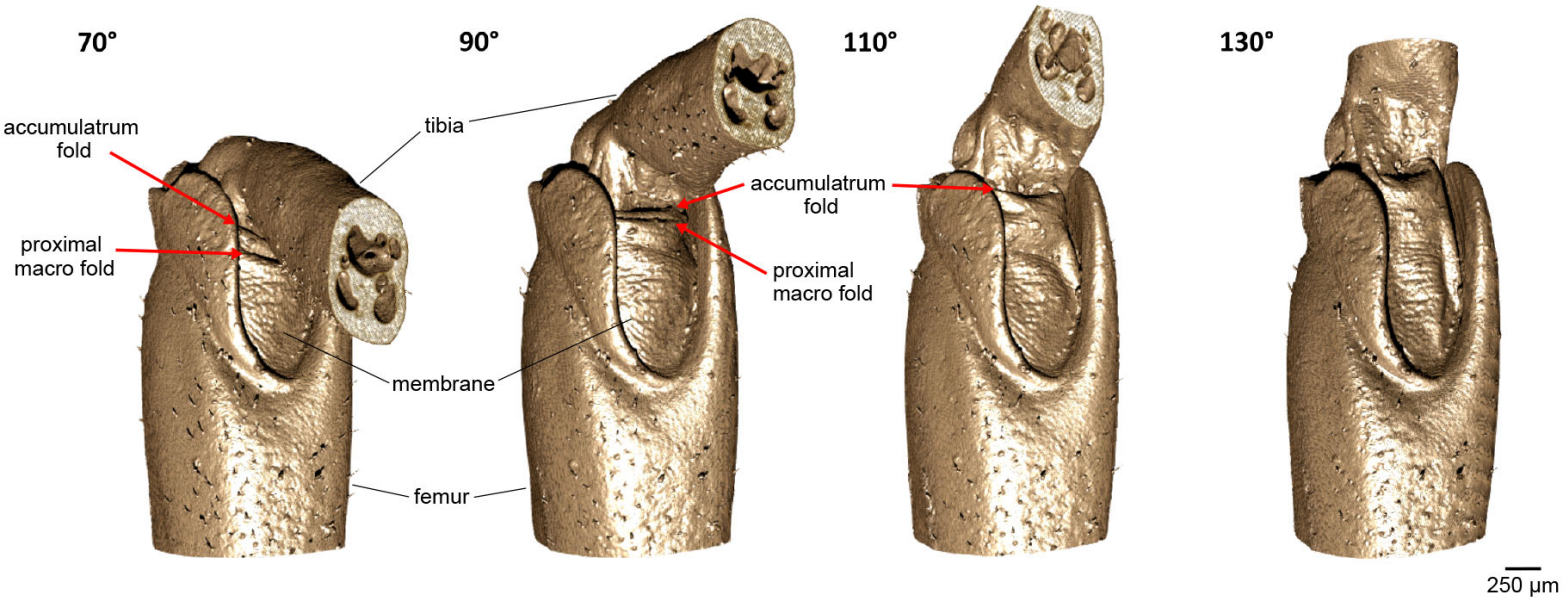
**Micro-CT scans at different joint positions (70° 90°, 110°, 130°) show the membrane folding in the femur–tibia joint in the mesothoracic legs of locusts with a voxel size of 6.37 µm.** In the bent joint positions (70° and 90°), two distinct folds can be obtained in the articular membrane: the accumulatrum fold and the proximal macro fold. In the stretched joint positions (110° and 130°), the proximal macro fold is no longer present. In addition, the pocket-like structure of the accumulatrum fold is visible at 110° and 130°.

To examine the shape and kinematics of the macrofolds, the leg joints were analysed at different joint positions using micro-CT (Fig. 1). The 3D reconstruction clearly shows two prominent macro folds in the distal, tibial sided area of the membrane at 70° joint angle (Fig. 2). These macro folds were present in freshly moulted adult insects as well as in fully mature adult insects.

When the joint is extended, the membrane elongates and the two macrofolds unfold until only the upper (distal) macrofold remains visible at around 110°. The lower (proximal) macrofold, visible at 70° and 90°, was less consistent across individuals. In contrast, the upper macrofold was consistently present and also has a characteristic kinematics depending on joint position. At joint angles of 110° and 130°, a round, pocket-shaped structure or ‘sink’ formed behind the upper macrofold, serving as a reservoir (*accumulatrum*) to store excess membrane. This distal macrofold will henceforth be referred to as the ‘accumulatrum fold’.

The kinematics of the accumulatrum fold suggest the presence of an internal rolling mechanism. In the flexed joint position, the fold is mostly rolled up, while it unfolds as the locust leg extends. Even at maximum extension during everyday locomotion, the accumulatrum fold is still visible.

**Fig. 3.**
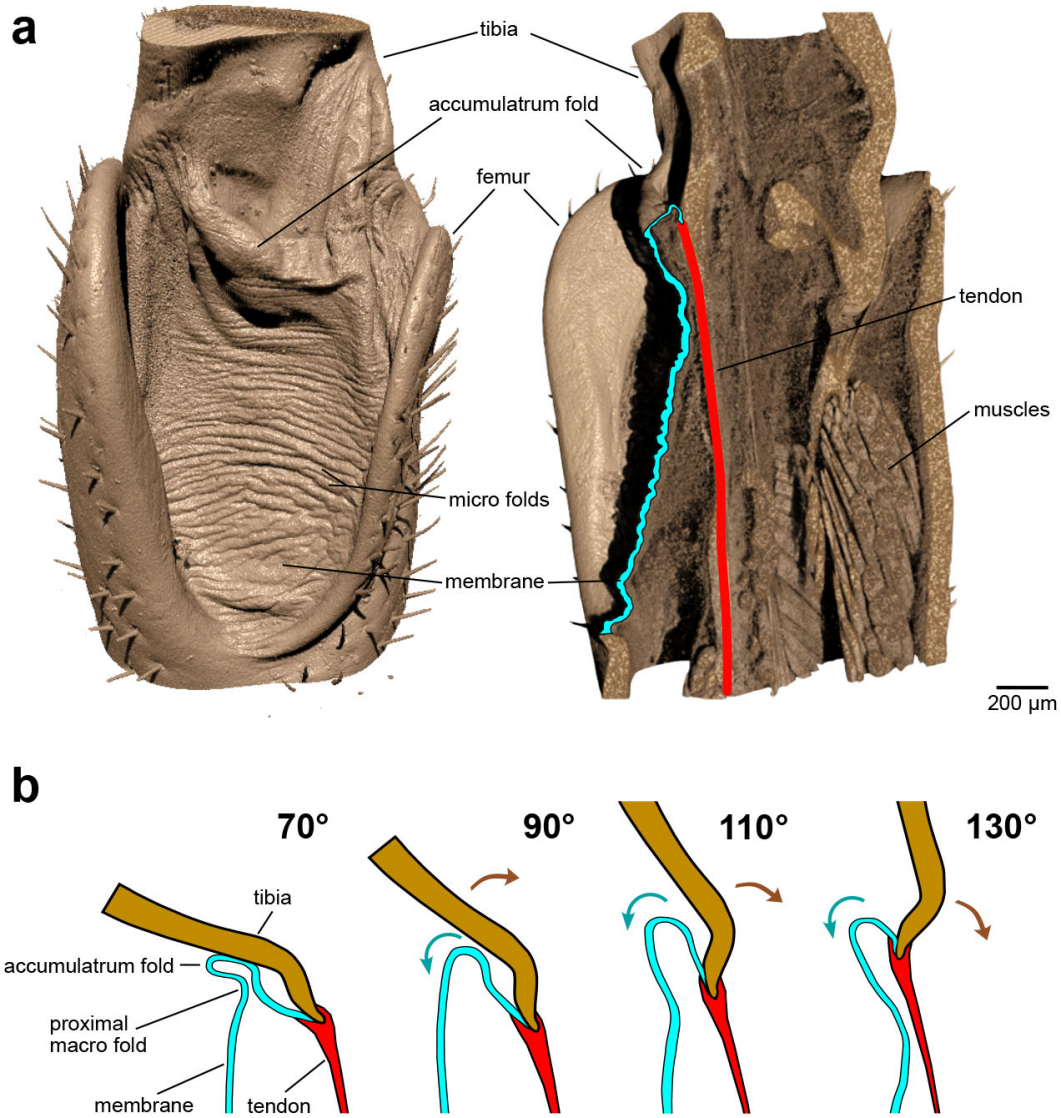
**Membrane folding and internal structures of the femur–tibia joint in the mesothoracic legs of locusts.** (A) X-ray microscope scans visualize not only the accumulatrum fold but also the numerous microfolds (voxel size: 2.67 µm) due to the significantly higher resolution. The sectional view (sagittal section) shows internal structures such as the tendon (marked in red) or muscles that affect the folding of the articular membrane (marked in blue). (B) Schematic illustration of the membrane folding that describes the unrolling of the accumulatrum fold when the leg is stretched. At 70° and in some cases also 90°, the tibia touches the accumulatrum fold, causing the formation of the proximal macro fold parallel to the accumulatrum fold.

Stained XRM scans (*n*=3) provided further detail on internal structures, such as muscles and tendons, as well as membrane thickness (Fig. 3A). The membrane folds onto to the apodeme (Foelix, 1979; Chapman et al., 2013) of the depressor muscle, forming the pocket-like structure. The flexor muscle also attaches to the apodeme within the sink of the accumulatrum fold, at the junction of the membrane and apodeme.

Folding kinematics during extension are illustrated in Fig. 3B. At a joint angle of 70°, the accumulatrum fold is fully rolled up but folds again upon contact with the ventral side of the tibia. No internal structures were identified that could favour or induce folding in the proximal macrofold. By contrast, the accumulatrum fold is formed through its connection to the apodeme and internal structures. As the leg extends, the flexor muscle elongates, causing the apodeme attachment point to shift upwards while the tibia rotates around the joint axis. This movement elongates the membrane and unrolls the accumulatrum fold. The primary fold axis of both macrofolds aligns nearly parallel to the joint's axis of rotation (Fig. 2).

### Shape and kinematics of the microfolds

**Fig. 4.**
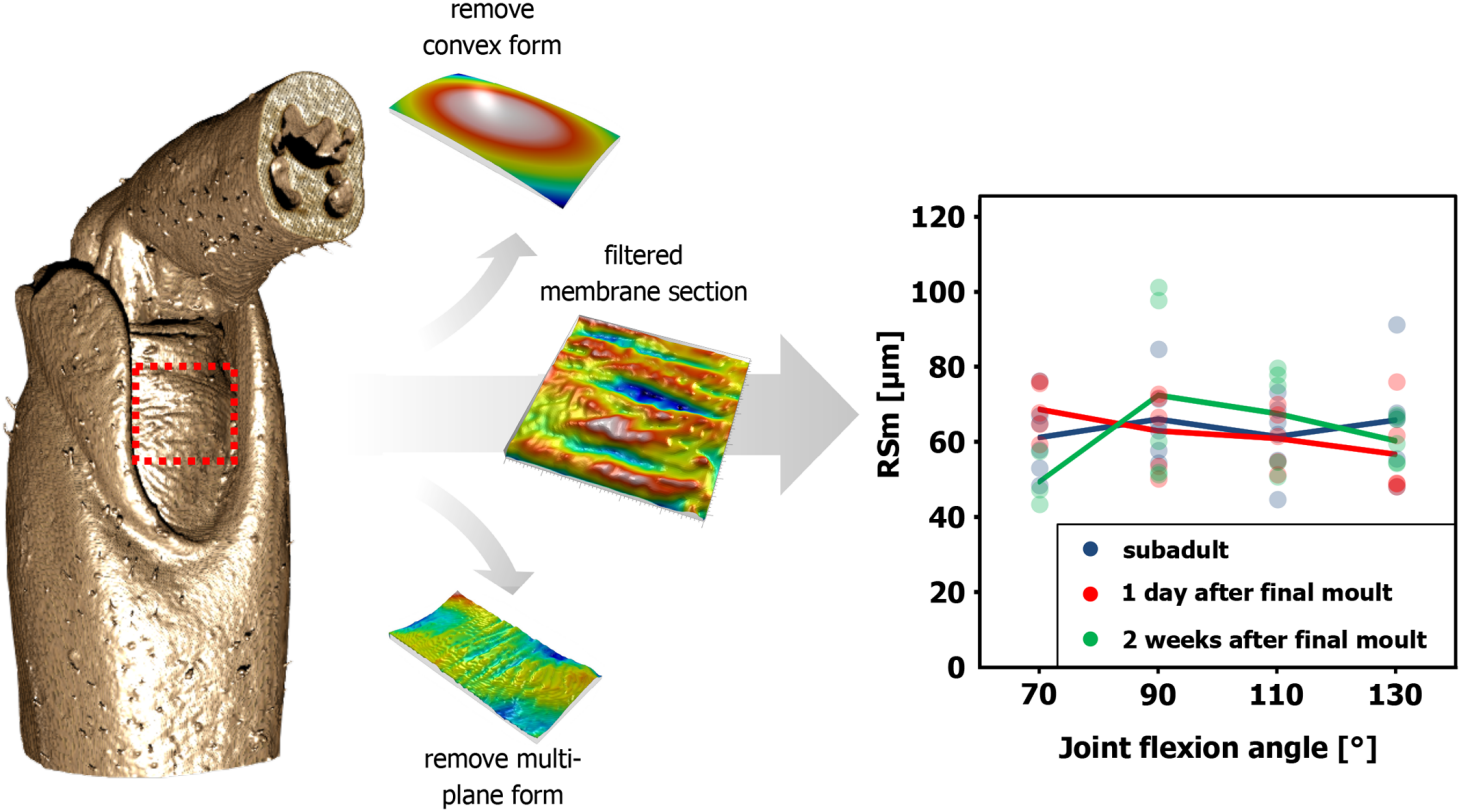
**Roughness analysis of the articular membrane.** Three-dimensional (3D) membrane sections were created in 3DSlicer from the CT scans and imported into MountainsMap in order to correct for the general convex shape of the membrane sections and additional macroscopic geometric irregularities [multi-plane form]. A roughness analysis was then performed on the filtered membrane sections according to the ISO 4287 standard. The diagram shows the average spacing of the microfolds (RSm) at different joint positions of the femur–tibia joint in the mesothoracic legs of subadult locusts (blue, *n*=5), freshly moulted locusts one day after final moult (red, *n*=5) and fully tanned locusts 2 weeks after final moult (green, *n*=6). Neither the developmental stage nor the joint position has any effect on the average spacing of the microfolds.

In addition to the prominent macrofolds, our CT scans revealed numerous fingerprint-like microfolds distributed across the entire articular membrane (Fig. 2). The microfolds are primarily arranged transverse to the leg, i.e. approximately parallel to the axis of rotation of the femur–tibia joint. At first glance, the microfolds appear to be much less specific than the larger accumulatrum fold. This is confirmed by higher-resolution XRM scans (Fig. 3A). Detailed analysis showed that the peak-to-peak spacing of microfolds ranges from 40 to 100 µm (Fig. 4). This pattern was not noticeably affected by either joint position or developmental stages.

To investigate the origin of microfolds, XRM data were analysed to consider material thickness and internal structures. Scans revealed that the membrane in the creases of microfolds was, on average, one-third thinner than at the peaks.

### Histology of the articular membrane

**Fig. 5.**
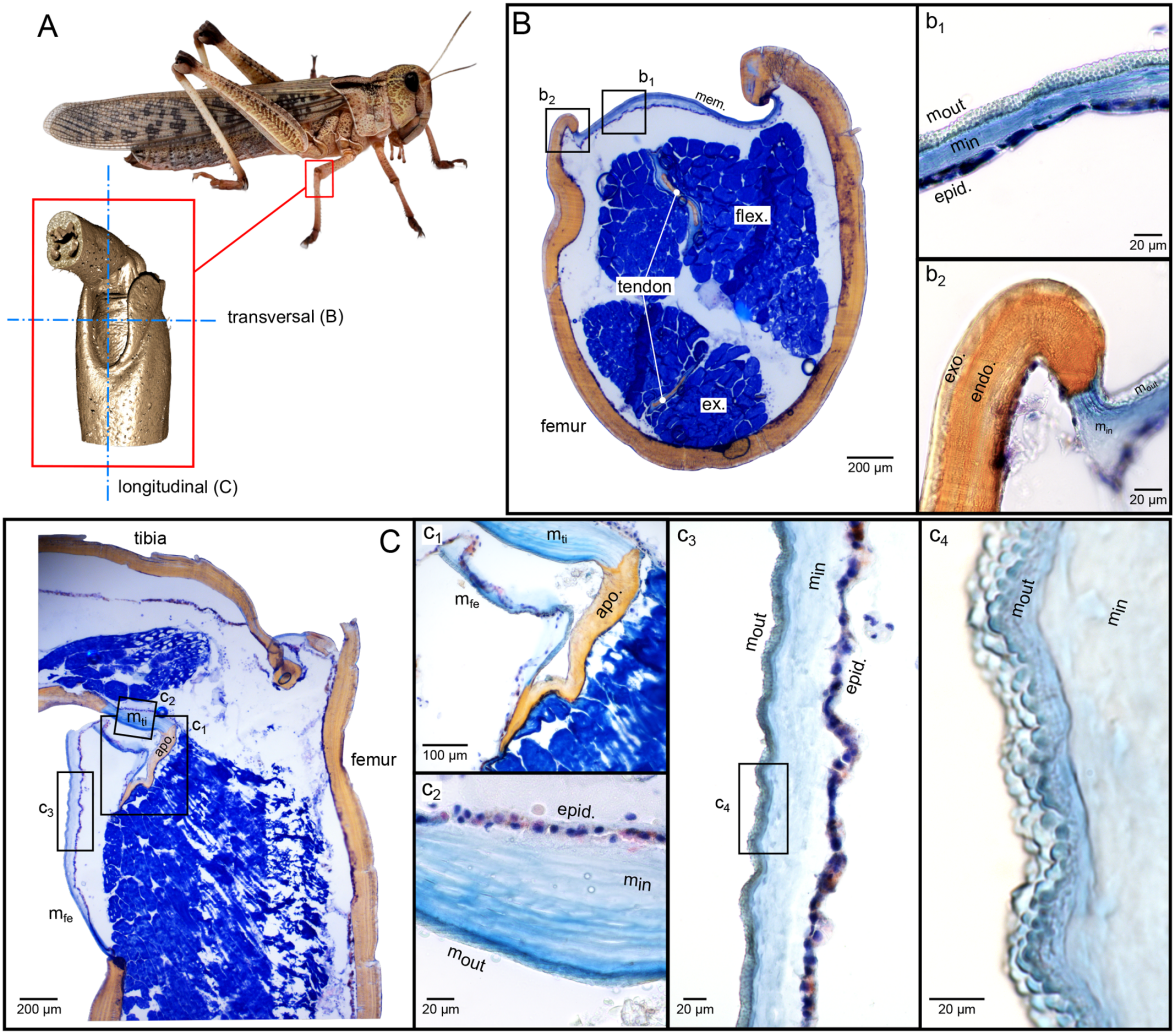
**Histological cryo-sections of the femur–tibia joint in the mesothoracic legs of locusts.** (A) a male *L. migratoria*, its femur–tibia joint in the mesothoracic leg is marked in a red box. The red box shows a CT scan of the femur–tibia joint with the transverse and longitudinal section planes of the cryosections. (B) Azan-stained transverse cryosection showing the orange-stained, sclerotized femur cuticle and the blue-stained, non-sclerotized articular membrane (*mem*). Within the femur, the non-sclerotized flexor muscle (*flex*) and extensor muscle (*ex*) with the respective tendons can be seen. (B,b1) Close-up of the articular membrane including epidermal layer (*epid*), inner membrane layer (*m_in_*) and outer membrane layer (*m_out_*). (B,b2) Close-up of the transition of the sclerotized femur cuticle, consisting of exocuticle (*exo*) and endocuticle (*endo*) to the non-sclerotized membrane. (C) Azan-stained longitudinal cryosection through the femur–tibia joint with (C,c1) Close-up of the apodeme (*apo*), which divides the articular membrane into a femoral (*m_fe_*) and tibial (*m_ti_*) membrane part. (C,c2) Close-up of the tibial membrane (*m_ti_*) consisting of epidermal layer (*epid.*), inner membrane layer (*m_in_*) and outer membrane layer (*m_out_*). (C,c3) Close-up of the femoral membrane (*m_fe_*) consisting of epidermal layer (*epid*), inner membrane layer (*m_in_*) and outer membrane layer (*m_out_*). (C,c4) Close-up of the surface structure and distinct differentiation of the outer and inner membrane layers of the femoral membrane. A white balance was performed manually using Gimp (version 2.10.36).

Light microscopy was used to analyse the histological composition of the articular membrane sections (*n*=3). Between 10 and 15 measurements per individual were made to determine layer thicknesses. Azan staining highlighted sclerotized elements of the insect cuticle in orange, while non-sclerotized components, such as the epidermal layer and muscles stain blue (Wang et al., 2019). Our results show that in neither longitudinal nor transversal cross-sections did the articular membrane react to Azan staining in the same way as the exocuticle and endocuticle of the femur or tibia, indicating a significantly lower or non-existent degree of sclerotization (Fig. 5).

A transverse section of the membrane (Fig. 5B) displays a dark-blue to violet-stained inner layer representing the epidermal layer (Fig. 5B,b1). Variations in the shades of orange within the femur cuticle (Fig. 5B,b2) reflect differing degrees of sclerotization. The outer cuticle layer, with a thickness ranging from 11 to 21 µm, is thinner than the inner cuticle layer, which ranges from 50 to 125 µm. Further layers are visible within the endocuticle, which most likely represent daily growth bands with different orientations of the chitin fibres (Neville, 1963a,b, 1967, 1983; Sviben et al., 2020). Blue-stained structures in the section correspond to the flexor and extensor muscles, with sclerotized tendons visible within them (Fig. 5B).

Both the hardened cuticle of the femur and tibia and the articular membrane exhibit a layered composition. The transition from the sclerotized femur cuticle to the membrane (Fig. 5b2) shows a gradual change in the exocuticle, which merges into the blue-stained, less sclerotized outer membrane layer. Conversely, the transition from the endocuticle to the membrane's inner layer is more abrupt. Interestingly, smaller layered structures, most likely daily growth bands, are evident at the endocuticle-inner layer interface. These layers are more prominent in Fig. 5b1 and under the polarization microscope (Fig. S1). Two distinct types of layers are visible: (1) lamellar layers extending throughout the endocuticle of the membrane (Fig. S1a), and (2) thin lamellar layers near the outer membrane surface (Fig. S1b).

The articular membrane stained blue in both transverse (Fig. 5B) and longitudinal (Fig. 5C) sections, confirming a much lower degree of sclerotization compared to other cuticular parts of the femur–tibia joint. The membrane's total thickness ranges from 24 to 69 µm, making it thinner than the femur or tibia cuticle, which ranges from 54 to 143 µm in representative individuals. Like the procuticle of the femur or tibia, the articular membrane consists of two distinct layers: a thinner outer layer (6.3 to 15.4 µm) and a thicker inner layer (12.5 to 63.2 µm).

The sclerotized apodeme (Fig. 5c1), located within the sink behind the accumulatrum fold, divides the membrane into tibial (Fig. 5c2) and femoral (Fig. 5c3,c4) regions. The tibial membrane, with a total thickness of 56 to 104 µm (outer layer: 7.8 to 10.9 µm; inner layer: 49 to 96 µm), is notably thicker than the femoral membrane and resembles the dimensions of the sclerotized tibial cuticle.

The surface structures show distinct differences between the tibial and femoral membranes. While the femoral membrane has tiny, rounded knobs (Fig. 5c4), the tibial membrane exhibits a much smoother surface (Fig. 5c2). In the tibial membrane's inner layer, distinct layers are visible, and polarization microscopy confirms these as daily growth bands (Fig. S1).

The transition between the tibial membrane and sclerotized tibial segments or tendons mirrors the femoral membrane's transition. The outer layer gradually merges into the membrane's outer layer, while the inner layer's transition is abrupt. Longitudinal sections (Fig. 5C) show the epidermis, outer and inner membrane layers, and transversely formed microfolds. Neither the creases nor the peaks of these microfolds displayed local sclerotization (Fig. 5c3). The contour of the microfolds is more pronounced on the external membrane surface than on the inner, epidermal side.

### Surface structure of the articular membrane

**Fig. 6.**
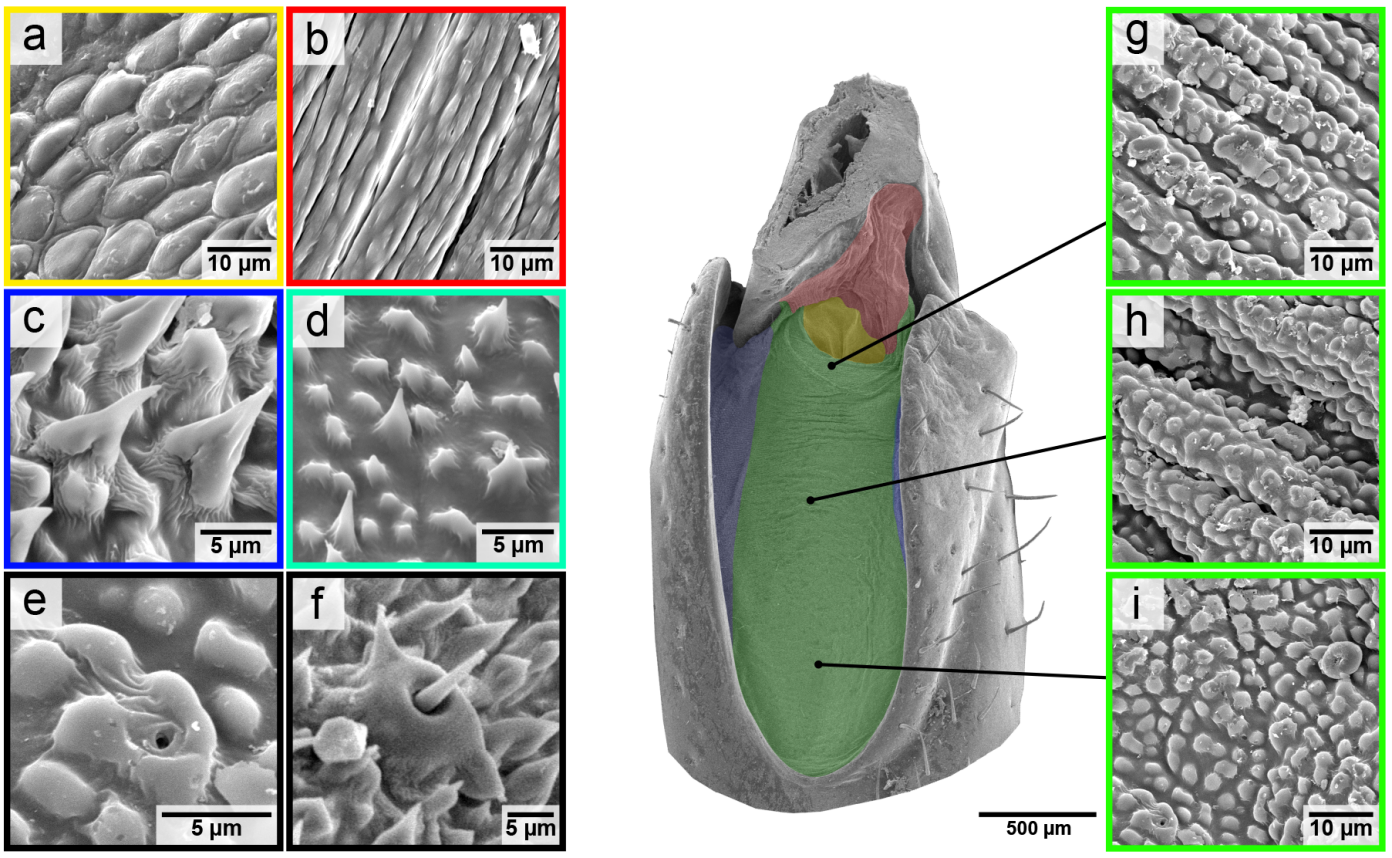
**SEM images of the diverse surface structures on the articular membrane.** This is a SEM image of the articular membrane of the femur–tibia joint in the mesothoracic legs of locusts. The membrane is divided into different areas by colour to show the presence of the different structures. The subfigures have a coloured frame that shows in which area the structure is found. (A) Scale-like surface structure of the apodeme behind the accumulatrum fold. (B) Flat, elongated or stretched knobs on the surface of the tibial membrane. (C) Spines in the area of the articular membrane that are overlapped by the femur cuticle. (D) Transition of the membrane area with spines (blue) and the membrane area with tiny, rounded knobs (green). (E) Pore channels that are distributed over the entire membrane. (F) Mechanosensors that occur in the green membrane area, but more frequently in the blue membrane area. (G-I) Numerous tiny, rounded knobs that cover the largest part of the membrane. The arrangement of the knobs varies. While the nobs in the area of the accumulatrum fold (G) seem to correlate with the microfolds, the nobs in the proximal membrane area (I) are distributed rather randomly.

The intricate surface structure of the articular membrane in the femur–tibia joint was analysed using SEM (Fig. 6). The results revealed a highly diverse surface, which could be divided into distinct regions characterized by unique structural features. The structures were measured on three representative adult, fully mature individuals, with at least 12 to 15 measurements per individual for each region with unique structural features.

The most prevalent feature of the membrane's surface is the presence of rounded knobs, which cover the largest area (Fig. 6G-I, green). These knobs measure 2.20±0.55 µm in diameter (*n*=3; 88 measurements), 1.31±0.62 µm in height (*n*=3; 49 measurements), and are spaced 1.06±0.54 µm apart (*n*=3; 77 measurements). While the knobs are distributed irregularly across the membrane (Fig. 6I), they align along the microfold peaks in the region of the accumulatrum fold and appear to correlate with folding patterns (Fig. 6G). Interestingly, knobs are absent in the creases of the microfolds, occurring only on the peaks.

Behind the accumulatrum fold, on the apodeme, a smoother, scale-like structure is observed (Fig. 6A, yellow). The individual scales measure 9.29±3.54 µm in diameter (*n*=3; 12 measurements), and a spacing of 1.20±0.54 µm (*n*=3; 12 measurements).

In the tibial membrane area, instead of transverse microfolds, longitudinal microfolds are present, which appear to correlate with a distinct surface structure (Fig. 6B, red). Here, the surface is characterized by flat elongated knobs, measuring 8.38±2.39 µm in length (*n*=3; 17 measurements) and 2.14±0.39 µm in width (*n*=3; 17 measurements).

Another unique region displays vertical spines (Fig. 6C, blue), with diameters of 5.55±0.62 µm (*n*=3; 15 measurements), heights of 7.71±1.59 µm (*n*=3; 12 measurements), and spacing of 4.29±1.03 µm (*n*=3; 14 measurements). The transition between spiny and knobbed areas is gradual (Fig. 6D), with pure spine regions localized to the medial side of the membrane.

Additionally, tiny pore channels (Fig. 6E) and mechanosensors (Fig. 6F) are scattered throughout most of the membrane, except for the apodeme surface behind the accumulatrum fold. Mechanosensors occur more frequently in spiny areas. All observed features: rounded knobs, scales, spines, pore channels and mechanosensors, were consistent across air-dried samples and samples subjected to critical point drying (including acetone treatment). These structures were also unaffected by mechanical cleaning (brushing and scraping with the back of a scalpel).

## DISCUSSION

Our findings demonstrate that the mobility of the femur–tibia joint in locusts is ensured by membrane folding rather than stretching. Two types of folds were identified: macrofolds with specific kinematics dependent on joint position and less specific microfolds, which are distributed over the entire membrane and whose mean spacing does not change depending on daily locomotion, age or usage. Histological analysis revealed no sclerotization in the membrane compared to the sclerotized cuticle of the femur and tibia. Additionally, SEM analysis highlighted distinct surface structures across the membrane. Below, we discuss the functional significance of these folds and their potential origins.

### Functional analysis of the folding

The accumulatrum fold, a distinct macrofold in the femur–tibia joint, was consistently observed across all samples, regardless of developmental stage or sex. This fold, formed by the attachment of the depressor muscle apodeme to the membrane, provides structural direction for controlled folding. Even at maximum leg extension, the fold's shape is maintained, preventing misfolding. This mechanism allows energy-efficient, resistance-free joint movement and controlled membrane storage during flexion, similar to wing folding in flying insects (Haas and Beutel, 2001; Faber et al., 2018). Controlled membrane storage avoids uncontrolled folds in unfavourable areas that could interfere with joint movement. A purely elastic membrane without folds, would require additional muscle force for stretching, compromising movement efficiency (Vincent, 1975; Göttler et al., 2021).

The proximal macrofold showed some variability across individuals, possibly due to differences in membrane biomechanics and tibia shape. Its formation may result from the accumulatrum fold colliding with the ventral side of the tibia compressing the membrane during flexion.

Microfolds, identified as secondary folds, are consistently present regardless of age, joint position (Fig. 4) or sample preparation, which means that the folds are not artifacts of drying or age-related changes. Vincent, 1981 also found numerous microfolds in the abdominal intersegmental membrane of female locusts and showed that the microfolds are formed early during the formation of the cuticle. The fact that the joint position also has no effect on the microfolds shows that during normal locomotion, most of the membrane displacement occurs through the specific accumulatrum fold. Accordingly, the microfolds could serve as a ‘material buffer’ for more extreme movements that would not yet result in muscle injuries but could lead to a failure of the membrane due to stretching (Vincent, 1981; Zhao et al., 2016). This aligns with Vincent's (1975, 1981) observations in abdominal intersegmental membranes of female locusts, where microfolds enable greater elongation without membrane rupture. Furthermore, microfolds may enhance flexibility in the accumulatrum fold (Politi et al., 2019), similar to the principle of an accordion, which is more flexible due to many folds. The hierarchical arrangement of macrofolds and microfolds could ensure controlled movement, improving energy efficiency and material durability (Göttler et al., 2021). However, micro-CT resolution may have limited detection of subtle microfold changes during joint movement.

### Histology of the articular membrane

Our histological staining results revealed significant sclerotization in the cuticle of the femur and tibia (Fig. 5). The apodeme, dividing the articular membrane into femoral and tibial parts, displayed similar sclerotization to the femur and tibia cuticle (Andersen, 1973). In contrast, the tibial and femoral membrane indicated a non-existent or at least notably lower degree of sclerotization, which is consistent with findings by Vincent (1981) on abdominal intersegmental membranes in locusts. The absence of locally sclerotized areas in transverse and longitudinal sections suggests that microfold patterns are not caused by material differences due to sclerotization.

Ultrastructural analysis showed that the femur and tibia cuticles consist of two distinct layers: a sclerotized outer exocuticle and a significantly thicker inner endocuticle (Fig. 5b2). The exocuticle (11.44–20.53 µm) and endocuticle (50.02–125.16 µm) thicknesses align with previously reported values for locust cuticles (Neville, 1965; Li et al., 2020; Stamm and Dirks, 2022). Although both layers are stained orange, the different shades of orange indicate a different degree of sclerotization. Since the outer exocuticle has a brownish to cream-coloured component, the sclerotization appears to be more intense here than in the endocuticle. In comparison to the exocuticle and endocuticle, the colouring of the membrane is clearly different, it is blue and therefore not sclerotized (Wang et al., 2019). The femoral membrane, ranging from 24–69 µm in thickness, is thinner than the femur and tibia cuticles, with the tibial membrane resembling the tibia cuticle dimensions (Fig. 5C). During joint movement, displacement predominantly occurs in the thinner femoral membrane area. The tibial membrane area is presumably significantly thicker and therefore more rigid, as the forces of the flexor muscle are transmitted to the tibia here and stretching of the membrane should be avoided in order to prevent the forces from partially dissipating in the stretching of the membrane. Nevertheless, this tibial membrane area is not sclerotized, possibly to remain movable and thus ensure an ideal, permanently adapted angle for force transfer (Chapman et al., 2013; Politi et al., 2019).

In the literature, it has previously been assumed that the intersegmental or articular membrane is only composed of endo- and epicuticle and that no exocuticle exists here (Dennell and Malek, 1954; Göttler et al., 2021). Vincent (1981) also found two main layers in the intersegmental membrane of female locusts. Similar to our study, Vincent (1981) also documented a layer thickness of approximately 15 µm for the outer layer of the membrane, whereby he documented a thickness of approximately 150 µm for the inner layer. Vincent (1981) describes the outer membrane layer as epicuticle and the inner layer as endocuticle. If we compare these layer thicknesses of the membrane from Vincent (1981) but also from our study with the layer thicknesses of locust cuticle documented in the literature (Neville, 1965; Li et al., 2020; Stamm and Dirks, 2022), it is noticeable that the epicuticle is usually in the nanoscale range with 510±20 nm (Li et al., 2020), whereby the layer thickness of the outer membrane layer (6-15 µm) corresponds very well with the layer thickness of exocuticle (12±3 µm) (Li et al., 2020; Stamm and Dirks, 2022). It is therefore possible that the outer layer of the membrane is not epicuticle, as Vincent (1981) suggests, but non-sclerotized exocuticle. Gorb (2001a,b) also describes that the membrane in the pretarsus of damselflies consists of an electron-dense, external, exocuticular layer and an electron-lucent, inner, endocuticular layer. The gradual transition from the femur exocuticle to the membrane's outer layer supports this interpretation (Fig. 5b2). The transition from sclerite to membrane shows the gradual characteristics of cuticle which can influence the formation of microfolds as well (Rajabi et al., 2017; Politi et al., 2019; Jafarpour et al., 2020).

Additionally, lamellae within the membrane, identified as daily growth bands, were visible under polarized light microscopy (Neville, 1963a,b, 1967, 1983; Sviben et al., 2020). These daily growth bands were detected in the entire endocuticle of the membrane (see Fig. S1a) and in a smaller area near the membrane surface (see Fig. S1b). Similarly, Vincent (1981) identified two types of lamellar layers in the abdominal intersegmental membrane of locusts: a thin layer (∼5 µm thick) of helicoidal layers directly underneath the outer membrane layer and a larger area of elastomer layers between extending to the epidermis. Göttler et al. (2021) also documented laminar layers in the endocuticle of jumping spider articular membranes. Despite these observations, our study did not find a direct correlation between lamellar layers and microfold formation.

Studies on jumping spiders (Göttler et al., 2021) and honeybees (Zhao et al., 2016) demonstrated that differences in membrane thickness locally adapt mechanical properties, enabling specific and controlled folding. This aligns with our observation that the articular membrane is thinner in microfold creases. Factors such as chitin fibre orientation, proteins, hydration, and surface structures may also contribute to microfold formation (Hackman and Goldberg, 1987; Tychsen and Vincent, 1976; Vincent, 2002).

### Surface structure of the articular membrane

Insect cuticle surfaces often exhibit diverse nano- and microscale structures with multifunctional properties. The surface structures of e.g. wings (Ma et al., 2019) or toepads (Dirks et al., 2012; Dirks, 2014) are well-documented, the surface structure of intersegmental or articular membranes remains less explored. Studies have identified rounded knobs on cockroach abdominal intersegmental membranes (Dennell and Malek, 1954) and distinct surface patterns on the articular membrane of jumping spiders (Göttler et al., 2021). The subdivision of the articular membrane into different areas with very different surface structures is described for the first time in this work (Fig. 6).

Surface structures can serve various functions, including antimicrobial activity, self-cleaning, adhesion, friction reduction, aerodynamics, defence, sound generation, thermoregulation, nutrient transport or sensory perception (Richards and Richards, 1979; Vincent and Wegst, 2004; Watson et al., 2017). Many of these functions, such as antimicrobial properties and light interactions, are unlikely here since they typically involve nanoscale structures, whereas the observed features are microscale. Adhesive structures such as those on the toepads of insects are also rather unlikely, considering the morphology of the structures but also the function of the joint to allow resistance-free movements (Dirks et al., 2012; Dirks, 2014).

The defence hypothesis seems the most plausible, as the articular membrane represents a mechanical weak point in the exoskeleton. Parasites, such as mites, often inhabit joint regions in locusts (Lawrence, 1940). Long, stiff spines or scales may also serve as defence for predators or parasites (Nutting and Spangler, 1969; Gross, 1993). We find spines in particular in the area where the cuticle of the femur overlaps the membrane (Fig. 6C) and scales in the sink behind the accumulatrum fold (Fig. 6A), in other words two areas in which mites could settle in a protected manner. This supports the assumption that these structures serve to defend against parasites and thus also ensure unhindered membrane folding. However, the knobs, which make up the largest part of the membrane surface (Fig. 6G-I), do not belong to structures that could serve as parasite defence.

Hydrophobic properties are another possible function. Hydrophobic surfaces can repel water, reducing accumulation in humid conditions or fog, which could otherwise hinder membrane folding through surface tension. The joints are also permanently exposed to contamination from dust and sand: a hydrophobic membrane surface would be an advantage for unrestricted joint movement due to possible self-cleaning effects (Wagner et al., 1996; Watson et al., 2017). Though hydrophobic structures typically range from 0.1 to 1.0 µm, and are thus significantly smaller than the surface structures observed here (Wagner et al., 1996). However, Bello et al. (2022) describe that microstructures, often in combination with nanostructures, can also have hydrophobic properties. It is possible that the knobs on the articular membrane of locusts are also covered with nanostructures, which we cannot visualize with our methods or due to the coating of the samples in the SEM.

The observed surface structures might also affect friction forces within the joint. Spines on stink bug wings, so-called microtrochia, for example, are associated with friction reduction by reducing contact area between sliding surfaces (Ma et al., 2019). The spines on the articular membrane of locusts (Fig. 6C) thus might also affect friction forces and folding of the membrane.

Controlling mechanical properties could also be a function of the surface structures. Göttler et al. (2021) identified surface structures on the articular membrane of jumping spiders, which correlate with mechanical properties and the folding pattern.

Improving the fatigue properties of the membrane due to surface structure is another conceivable feature of the structures. The knobs on the articulated membrane of locusts (Fig. 6G-I) could control the maximum curvature of the membrane with the diameter and spacing of the knobs, similar to the principle of a pearl necklace. Due to the diameter and spacing of the pearls, a pearl necklace can only be bent to a certain degree. Similarly, critical buckling in the membrane would then be avoided by the spacing and geometry of the surface structures, therefore also improving fatigue properties of the membrane (Watson et al., 2017; Göttler et al., 2021).

In summary, the described cuticular surface structures within the locust articular leg membrane could support and control membrane folding in many interesting ways. However, whether these structures actually form the microfolds remains unclear at present.

### Conclusion

Our work shows that the mobility of the femur–tibia joints of *Locusta migratoria* is not ensured by stretching but by folding of the joint membrane. Our studies have identified two different types of folds in the membrane: a specific macrofold (accumulatrum fold), which guides the major membrane movement, and less specific and much smaller microfolds, which could potentially function as material buffers at extreme joint positions. While the macrofold is formed by the connection to the apodeme and creates a reservoir, in which the membrane can be folded and stored in a controlled manner depending on the joint position, the formation of the microfolds is not yet fully understood. A locally adapted degree of sclerotization could be excluded as a reason for the formation of microfolds. The microfolds are also not formed by ageing processes or usage. It is possible that the microfolds in the membrane are formed by local differences in thickness or to the ultrastructure of the membrane, such as day/night layers. We were also able to identify different regions within the membrane with various characteristic surface structures. These complex surface structures could be relevant for the movement and possibly also for the mechanical properties of the membrane and therefore also be crucial for the formation of the microfolds and the membrane folding in general. However, the surface structures could also have other or additional functions such as parasite defence, self-cleaning properties or friction reduction to other surfaces to enable unfolding without resistance, to name just a few.

It therefore remains unknown how the microfolds are formed and what functions the diverse surface structures of the membrane have. Future studies should therefore focus on the formation and functional properties of the microfolds and the surface structure in order to further understand their influence on joint mechanics and durability.

## MATERIAL AND METHODS

### Sample preparation

Male and female migratory locusts (*Locusta migratoria*, Reptilienkosmos, Viersen, Germany) were bought at fifth instar stage. The animals were kept in groups of up to 30 specimens at room temperature (21.5±0.5°C) and a humidity of 61±4% with a day:night cycle of 12 h: 12 h. The insects were fed fresh food *ad libitum* (dried cereals, fresh carrots and fresh grass). For the studies locusts were sampled at three different stages: (1) subadult animals before the final moult; (2) freshly moulted animals, a maximum of 24 h after final moult and (3) adult animals 2 weeks after the final moult, with completely matured cuticle (Andersen, 1973; Neville, 1983). As the hind legs and in particular the femur–tibial joints are highly specialized for jumping, this study focused on the middle legs (mesothoracic) as representative samples for ‘walking legs’ (Fig. 1). The femur–tibia joints of the legs (right and left) were separated from froze insects (−20°C) right before the tests with a scalpel at the trochanter–femur joint and at the tibia approximately 3 mm distal to the femur–tibia joint. The samples were sealed with dental wax during the scan to reduce water loss and leakage of haemolymph. Locusts with visible damages or deformities were excluded from the experiments. The experiments were in full agreement with the German and European animal protection law. The authors strictly followed ethical guidelines to replace and reduce the number of animal specimens and refine experimental methods wherever possible.

### MicroCT and XRM analysis

To analyse the general external and internal morphology of the femur–tiba joint, scans were performed with the desktop X-ray microtomograph Skyscan 1275 (6.37 µm voxel size, 40 kV, 60 µA, 170 ms exposure time, 2× binning, Bruker Corporation, MA, USA). The femur–tibia joints of the middle legs of different stages were scanned at defined joint flexion angles of 70°, 90°, 110° and 130° using a custom-made sample holder made of acrylic glass (width: 10 mm, height: 20 mm, depth: 2 mm; Fig. 1). For each developmental stage, five to six middle legs from different individuals were scanned at each of the defined joint flexion angles. This angular range corresponds to the normal use of the mesothoracic leg during walking with 90° corresponding to the joint position in resting mode (Burns, 1972). To compensate for any possible remaining desiccation effects of the membrane during the scan time (a maximum of 90 min) the order in which the respective angles were scanned was randomized. For high-resolution scans with better contrast and a larger field of view of the intersegmental membrane we used a state-of-the-art X-ray microscope (2.67 µm voxel size, 80 kV, 80 µA, 2 ms exposure time, 90 min scan time, X-radia Versa 520, Zeiss, Oberkochen, Germany). For these scans the samples (*n*=3) were stained following an established protocol (Stamm and Dirks, 2022) and scanned in an Eppendorfer tube in 99.5% methanol.

All CT and XRM data were initially analysed using CTvox version 3.3.0.0 (Bruker Corporation, MA, USA) and CT Analyser v1.20.8.0 (Bruker Corporation, MA, USA) to perform a post alignment, a cropping of the volume of interest and to perform a thresholding to remove the grey values of the surrounding medium. A specialized ISO-certified surface texture software (MountainsMap version 8.2, Digital Surf, Besançon, France) was used to further quantify the folding patterns (Fig. 3). For the analysis 500×500 µm sections were manually selected from the centre of each articular membrane. Analyses of surface structures are made in reference to a flat reference, this is why the global form or shape of a surface has to be isolated or removed. To do this, the sections of the membrane data were filtered to correct for the nominal form, which is the general convex curvature of the sections due to the legs’ tubular geometry. This was done by the ‘form removal’-operator in Mountainsmap. In addition to the general convex shape of the membrane, however, there are also smaller curvatures and irregularities that were removed in a second step with the ‘remove Multi plane form’ operator with a 6th degree polynomial. Profile curves (north to south) were created at 25%, 50% and 75% of the width of the membrane section. For each of these profile curves, a 2D roughness analysis according to the ISO 4287 standard was performed. To quantify the average spacing of the microfolds the parameter RSm (average peak-to-peak spacing) was extracted.

### Histological sections and microscopy

A digital microscope (Keyence VHX-6000, Keyence Corporation, Osaka, Japan) was used to further check the folding-pattern and sclerotization of intact and complete articular membranes. The samples were examined immediately after preparation for a few minutes and therefore drying effects of the membrane are negligible. Overview images were taken at a joint flexion angle of 90° from the dorsal of the joints using the ‘Multilighting’ setting. To further analyse the composition of the membrane, histological sections of the femur–tibia joints were cut transversely and longitudinally to the leg axis using a cryomicrotome (12 µm, Leica CM1900, Leica Microsystems GmbH, Wetzlar, Germany). To visualize sclerotized areas of the cuticle the samples (*n*=3) were then azan stained and fixed according to Heidenhain (Romeis, 1989) and Dirks and Dürr (2011) without the xylene and alcohol series. The stained sections were analysed with polarization light microscopy (Nikon, BR 5043/1-1) using brightfield imaging to visualize cuticle layers according to Neville (1983; Stamm and Dirks, 2022). A white balance was performed manually using Gimp (V 2.10.36).

For SEM images of the membrane, the femur–tibia joints of the midlegs were critical point-dried (Leica EM CPD 300, Leica Microsystems GmbH, Wetzlar, Germany) and sputter-coated with gold (6-8 nm, Leica EM ACE600, Leica Microsystems GmbH, Wetzlar, Germany). The surface structure of the membrane was then analysed for three representative individuals using a Jeol JSM-6510 Series SEM (10 kV, JEOL GmbH, Freising, Germany).

## Notes

This text was improved for readability with the assistance of an AI language model (ChatGPT, OpenAI, 2024). After Using this tool, the authors reviewed and edited the content as needed and take fully responsibility for the content of the publication.

## Supplementary Material

10.1242/biolopen.061934_sup1 Supplementary information
