# Supplementary material for: Cuticular microstructure of the locust femur–tibia joint
Source: Biol Open. 2025 Jul 31;14(7):bio061934. doi: 10.1242/bio.061934 (PMC12352281; doi:10.1242/bio.061934)
Supplement: Supplementary information [file biolopen-14-061934-s1.pdf]

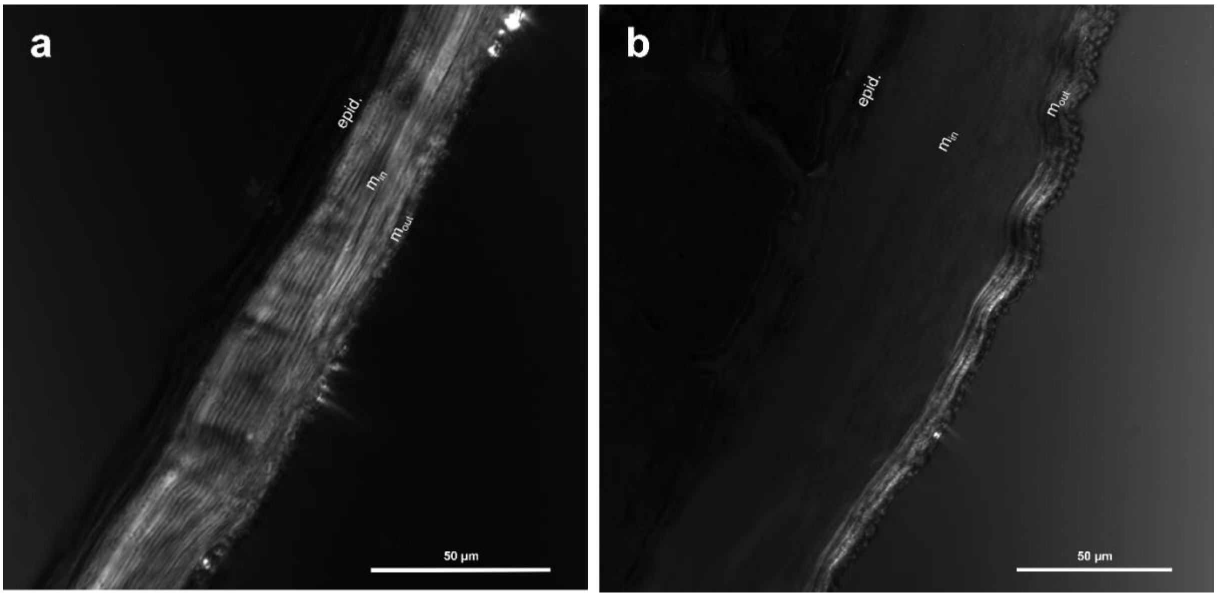


**Fig. S1. Daily growth bands in the articular membrane.** Histological sections show numerous lamellae within the articular membrane using the polarization microscope, which are daily growth bands with different orientation of the chitin fibers (Neville, 1963a; Neville, 1963b; Neville, 1967; Neville, 1983; Sviben et al., 2020). A) daily growth bands in the entire endocuticle of the membrane. B) A notably smaller area of daily growth bands in the outer membrane layer.

Biology Open • Supplementary information

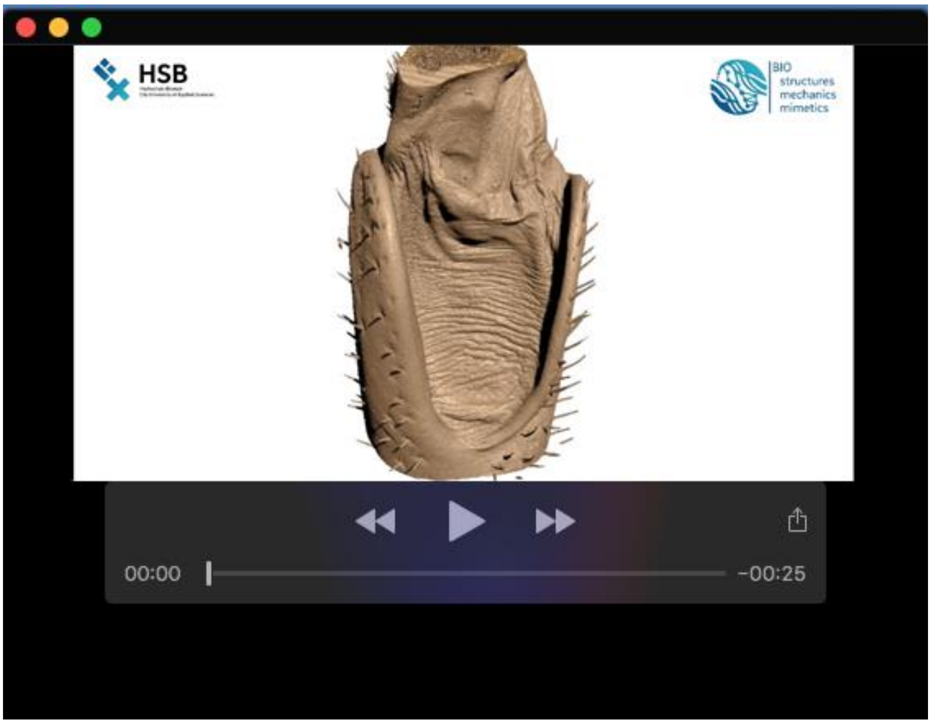


**Movie 1.** Video of the XRM-Scan of the femur-tibia joint in the right mesothoracic leg of an adult (2 weeks after final moult) female Locusta migratoria (voxel size: 2.67 µm) with sectional view.

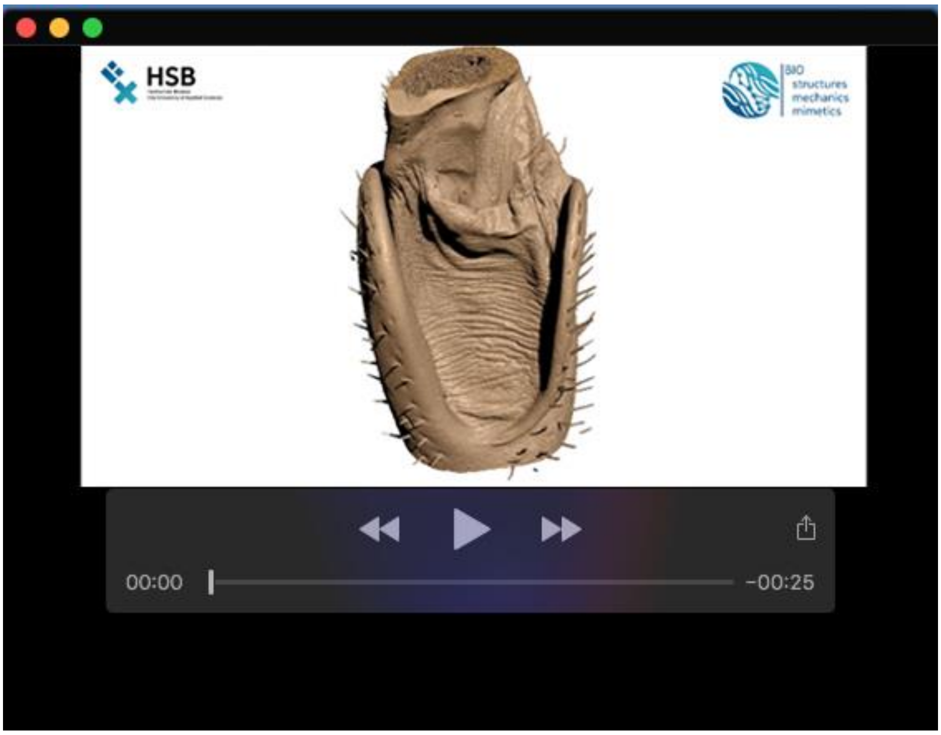


**Movie 2.** Video of the XRM-Scan of the femur-tibia joint in the right mesothoracic leg of an adult (2 weeks after final moult) female Locusta migratoria (voxel size: 2.67 µm) with 360°-view.
